# Diagnosis and treatment of IgA nephropathy and IgA vasculitis nephritis in Chinese children

**DOI:** 10.1007/s00467-022-05798-6

**Published:** 2022-11-08

**Authors:** Xuhui Zhong, Jie Ding

**Affiliations:** Department of Pediatrics, Peking University First Hospital, Beijing, China

**Keywords:** IgA nephropathy, IgA vasculitis-associated nephritis, Children, Chinese, Epidemiology, Kidney pathology, Treatment

## Abstract

IgA nephropathy (IgAN) and IgA vasculitis-associated nephritis (IgAVN) are among the most frequent childhood glomerular diseases and are characterized by significant variability in clinical manifestations, pathological presentation and long-term outcomes. IgAVN, alternatively called purpura nephritis, is pathologically indistinguishable from kidney-limited IgAN. In Chinese children, the clinical presentations and pathological manifestations of IgAN and IgAVN are variable. The severity of proteinuria and abnormalities in kidney function and blood pressure of children in China are comparable to those of children in Europe, the USA, and Japan. Compared to Caucasian children and Japanese children, crescents were more common in Chinese children with IgAN or IgAVN. Approximately 10–20% of childhood IgAN or IgAVN progresses to impaired kidney function in China. Since 2007, a series of guidelines on the diagnosis and treatment of pediatric kidney diseases has been published following the principles of evidence-based medicine. However, a large difference exists between the Chinese evidence-based guidelines and the guidelines developed by Kidney Disease: Improving Global Outcomes (KDIGO) in 2021. Chinese children with IgAN or IgAVN were more likely to be treated with steroids or immunosuppressive agents. Further studies exploring the optimal treatment regimen for childhood IgAN or IgAVN are needed in the future.

**Supplementary information:**

The online version contains supplementary material available at 10.1007/s00467-022-05798-6.

Immunoglobulin A nephropathy (IgAN) is one of the most frequent glomerular diseases in children and adolescents. It has been reported in 11.2% and 51.2% of kidney biopsies in Chinese children, by studies of 33 hospitals and national hospitalization data, respectively [1, 2]. Additionally, it has been detected in more than 25% of kidney biopsies from Japanese children [3] and in 20% of European children with biopsy-proven glomerular diseases [4, 5]. IgAN is defined as the predominant deposition of IgA in the glomerulus, as determined by immunofluorescence staining. The most common type of vasculitis in children is IgA vasculitis (IgAV) [6], which is also known as Henoch–Schönlein purpura. Children with IgAV might develop purpura, joint pain, gastrointestinal tract symptoms, and kidney involvement. IgA vasculitis-associated nephritis (IgAVN), alternatively called purpura nephritis, is pathologically indistinguishable from kidney-limited IgAN. Both IgAN and IgAVN are characterized by significant variability in clinical manifestations, pathological presentations, and long-term outcomes. Children present with variable clinical phenotypes that range from isolated hematuria to rapidly progressive glomerulonephritis. The pathological findings range from mild mesangial proliferation to crescentic formation. Regarding the long-term prognosis, approximately 10–20% of Chinese children with IgAN or IgAVN ultimately progress to kidney failure during young adulthood [7–10].

IgAN has a wide range of epidemiological characteristics, clinical presentations, disease development processes, and long-term prognoses across various racial groups [11]. Previous reports have documented demographic factors that increase the incidence of IgAN in young adult Caucasian and Asian patients [12, 13]. IgAN is more common and more likely to induce kidney failure in patients from East Asia [11]. Additionally, substantial differences have been found between adults and children with IgAN. It is unclear whether IgAN is two different diseases or the same disease in adults and children [14]. We also do not know if the above diversity is caused by heterogeneity in pathogenic, genetic, or environmental influences. Different manifestations of IgAVN have been documented in Chinese adults and children, with the involvement of the kidney being more frequent and severe in adults with IgAV [15]. However, further available evidence is extremely limited.

There are currently not enough data comparing the treatment efficacies between pediatric IgAN and IgAVN patients. Limited evidence exists to support treatment strategies in children. The guidelines developed by Kidney Disease: Improving Global Outcomes (KDIGO) [11] and Chinese evidenced-based guideline for children [16, 17] are quite different regarding the choice of steroids or immunosuppressive agents. Treatment practices for childhood IgAN and IgAVN vary greatly among different regions.

Therefore, both childhood IgAN and IgAVN exhibit great diversity in disease prevalence, manifestation, and prognosis across different regions. There is a pressing need to generate evidence for age-specific or race-specific diagnosis and treatment of these patients. Here, we will focus on the epidemiology, clinical manifestations, pathological findings, and evidence-based guidelines associated with Chinese children with IgAN and IgAVN. This could increase our understanding of disease phenotypes and improve clinical practices in China.

## Epidemiology of IgAN and IgAVN in Chinese children

According to a small sample study from Hong Kong, children below 16 years of age accounted for 11% of all Chinese patients with IgAN [18]. A nationwide survey was conducted retrospectively among hospitalized children from 1995 to 2004 in China. This survey demonstrated that IgAN was responsible for 1.37% of all hospitalized children with kidney diseases in China [1]. Other studies have explored the etiology of abnormal urinalyses in ‘healthy’ children [19, 20]. The percentages of IgAN and IgAVN were 10.6% and 1.7%, respectively, in children with abnormal urinalyses who were 6–15 years of age. IgAN was one of the major causes of abnormal urinalyses in children with isolated microscopic hematuria or microscopic hematuria with mild proteinuria.

In recent years, researchers have reviewed the kidney biopsy data from different areas in China. The proportion of IgAN or IgAVN in children who underwent native kidney biopsies ranged from 11 to 25% and from 4 to 16%, respectively [1, 21–23]. In some investigations, IgAN constituted the most common cause of primary glomerulonephritis in children, accounting for 16~36% of cases [21–23]. IgAVN was the predominant cause in 61–73% of children with secondary glomerulonephritis, such as lupus nephritis, hepatitis B virus (HBV) infection-related glomerulonephritis, and other ailments [24, 25]. IgAN and IgAVN are the key components of chronic kidney diseases in Chinese children. Disease distribution varies among different centers in China.

Data from other regions showed that IgAN accounted for more than 25% of kidney biopsies from Japanese children [3] and 20% of European children with biopsy-proven glomerular diseases [4, 5]. IgAVN is still the most prevalent cause of secondary glomerulonephritis, including lupus nephritis and HBV infection-related glomerulonephritis, as it is detected in nearly 20% of Japanese children [3] and 11.6% of Italian children [26]. A lower proportion of IgAN or IgAVN was found in biopsies from Chinese children (see Supplementary Tables 1 and 2); this might be attributed to ethnic disparities or indications of biopsy.

## Clinicopathological characteristics of IgAN in Chinese children

Two nationwide investigations were conducted retrospectively in 2004 and 2013 in China [1, 27]. IgAN was determined to be more predominant in males. The age at disease onset was 8.7 ± 3.3 and 10.0 (range 0.5–18.0) years of age, respectively. According to data from children hospitalized from 1995 to 2004 [1], isolated hematuria was the most common clinical manifestation. Isolated hematuria was found in 41.2% of these patients (including recurrent macroscopic hematuria in 26.9%), and this manifestation was followed by nephrotic syndrome (23.8%) and hematuria with proteinuria (20.8%). The proportion of rapidly progressive glomerulonephritis was 1.3%. In the 2013 report [27], the most prevalent manifestation was hematuria with proteinuria. Hematuria with proteinuria and nephrotic syndrome showed more prevalence in children with IgAN in the 2013 report, compared to the 2004 report (37.0% and 30.6% vs, 20.8% and 23.8%, respectively). The prevalence of isolated hematuria decreased from 41.2 to 15.8%. However, the prevalence of rapidly progressive glomerulonephritis was similar between the 2013 report and the 2004 report (1.3%). The above differences might reflect the changing trends of the clinical phenotypes of IgAN in Chinese children over time. It may also be affected by sampling bias or indication for kidney biopsy.

At present, the Oxford classification MEST-C scoring system has been widely applied to evaluate the kidney pathology of IgAN in China (Table 1). In 2012, Le et al. [7] collected information on 218 children with IgAN from seven Chinese kidney facilities. The proportions of cases with mesangial proliferation (M1), endocapillary proliferation (E1), segmental sclerosis/adhesion lesion (S1), moderate/severe tubular atrophy/interstitial fibrosis (T1/T2), and crescent formation (C1/C2) were 45%, 23%, 62%, 6%/1%, and 43.1%/0.5%, respectively. Twenty-four children (12.4%) developed kidney failure or a 50% reduction in kidney function after a median follow-up of 56 months. In Chinese children with IgAN, tubular atrophy/interstitial fibrosis was verified as the sole trait that was independently linked with poor kidney outcomes (HR 2.9, 95% CI 1.0–7.9, *P *= 0.04). Wu et al. [8] reviewed 1243 children diagnosed with IgAN from 2000 to 2017. The percentages of M1, E1, S1, T1/T2, and C1/C2 were 29%, 35%, 37%, 23%/4.3%, and 44%/4.6%, respectively. The multivariate Cox regression model indicated that lesions S (HR 2.7, 95% CI 1.8 ~ 4.2, *P* < 0.001) and T (HR 6.6, 95% CI 3.9 ~ 11.3, *P *< 0.001) might be predictors for poor prognosis. Crescent formation showed a significant predictive value only in children without immunosuppressive treatment. In another retrospective investigation of 98 children with IgAN [28], M1, E1, S1, T1/T2, and C1/C2 were identified in 42.9%, 70.4%, 18.4%, 21.4%, and 65.3% of cases, respectively. Mesangial C3 deposition ≥ 2 + combined with hypocomplementemia (C3 < 90 mg/dl) was an independent risk factor for a 30% decline in eGFR or kidney failure during follow-up. Therefore, the Oxford classification has a potential role in the prediction of kidney prognosis. Further study is still needed to validate the MEST-C scoring system in Chinese children with IgAN.

**Table 1:** Clinical findings at biopsy and histopathological characteristics according to Oxford classification of IgAN and IgAVN in children

Reference	N	Diagnosis	Age years old	Male %	Race/ethnicity	eGFR ml/min/1.73m^2^	Proteinuria	MAP mmHg	M1 %	E1 %	S1 %	T1/T2 %	C1/C2 %
[7]	218	IgAN	14.0 (range 2.0–17.9)	65.0	Chinese	134 ± 42	1.5 (range 0.5–8.0) g/day	88 ± 11	45	23	62	7	44
[28]	98	IgAN	9.7 (IQR 6.6, 12.2)	67.3	Chinese	108.5 ± 37.1	27.6 (IQR 11.5, 83.2) mg/kg/day	83 (IQR 79, 89)	42.9	70.4	18.4	21.4	65.3
[29]	90	IgAN	8.4 ± 3.2^a^	71.1	Chinese	142.6 ± 38.9	129.3 ± 64.9 mg/m^2^/h	NA	97.8	6.7	47.8	4.4	25.5
[8]	1243	IgAN	14 ± 4	68.0	Chinese	102 ± 20	0.6 (IQR 0.3, 1.4) g/day/1.73m^2^	89 ± 16	29.0	35.0	37.0	27.3	48.6
[9]	1243	IgAN	13.7 ± 3.7	67.7	Chinese	102.1 ± 19.8	1.0 (IQR 0.5, 2.4) g/day	89.4 ± 16.1	29.0	34.7	36.5	26.8	48.2
[30]	142	IgAN	10.1 ± 3.0	59.2	Chinese	109.4 (IQR 88.8, 125.8)	28.5 (IQR 11.9, 82.0) mg/kg/day	NA	98.5	78.7	17	18.4	70.9
[31]	1060	IgAN	12.7 (IQR 9.6, 15.4)	64.8	Caucasian Japanese Chinese Other	98 (IQR 79, 118)	1.2 (IQR 0.5, 3.0) g/day/1.73m^2^	85.1 (IQR 77.3, 92.9)	52.0	39.2	51.0	14.8	43.1
[4, 5]	174	IgAN	12.7 ± 3.6	71.8	European	117.0 (IQR 96.2, 120.0)	0.8 (IQR 0.3, 2.2) g/day/1.73m^2^	87.5 ± 11.4	21.8	13.8	42.5	6.3	14.9
[32]	161	IgAN	11.7 (range 3.6–19.4)	63.0	Japanese	103 ± 30	0.7 (range 0.0–13.7) g/day/1.73m^2^	79 ± 11	0.49^b^	0.8^b^	13.1^b^	3.3^b^	9.2^b^
[30]	57	IgAVN	10.2 ± 2.6	52.6	Chinese	114.2 (IQR 94.4, 131.1)	24.2 (IQR 9.3, 60.8) mg/kg/day	NA	92.7	81.8	23.6	10.9	80
[10]	104	IgAVN	10 (range 4–17)	56.0	Chinese	161 ± 48	1.7 (range 0.1–10.8) g/day	82 ± 11	66	56	62	58	61

*MAP*, mean arterial pressure; *eGFR*, estimate glomerular filtration rate; *NA*, not available; *IQR*, interquartile range; *M1*, mesangial proliferation; *E1*, endocapillary proliferation; *S1*, segmental sclerosis/adhesion lesion; *T1/T2*, moderate/severe tubular atrophy/interstitial fibrosis

^a^Age at disease onset

^b^The Oxford derivation cohort included children with proteinuria ≥ 0.5 g/day/1.73 m^2^

In general, the severity of proteinuria, average eGFR, and mean arterial blood pressure at biopsy in Chinese children with IgAN are comparable to that in children in Europe, the USA, and Japan (Table 1 and Supplementary Tables 1 and 2) [4, 5, 32–35]. Crescents were substantially more frequent in Chinese (45.7%) and Japanese (65.9%) children with IgAN than in Caucasian (25.5%) children with IgAN, according to an international cohort of children with IgAN [31].

## Clinicopathological characteristics of IgAVN in Chinese children

In China, a higher incidence of IgAV has been observed in the spring (27.4%) and winter (33.9%) seasons [36]. After the onset of IgAV, 96.7% of kidney involvement occurred within 6 months [37]. Children experiencing digestive symptoms were more likely to experience IgAVN [36]. Mao et al. [38] reported that at an age ≥ 6 years, purpura on the upper limbs or face and occult blood in the stool were all risk factors for the development of nephritis.

Clinical presentations and pathological manifestations of IgAVN were variable in Chinese children. A national retrospective survey was performed in 40 hospitals and included 4863 children diagnosed with IgAVN [37]. The incidence of IgAVN increased from 2008 to 2011. The male to female ratio was 1.52:1, and the mean age was 8.9 years. Hematuria with proteinuria was the most frequent clinical finding. This phenotype was found in 58.2% of patients and was followed by nephrotic syndrome (13.8%) and isolated hematuria (13.2%). Rapidly progressive glomerulonephritis was less common (0.2%) than IgAN. Male patients were more likely to have proteinuria.

In China, the ISKDC classification (International Study of Kidney Disease in Children) [39] system is extensively used for the pathological diagnosis of IgAVN in children. The most prevalent pathogenic types were subclasses II (31.0%) and III (53.5%) [37]. In general, crescent, segmental glomerulosclerosis, glomerular capsule adhesion, and endocapillary proliferation were related to clinical symptoms in children with IgAVN [40]. For example, pediatric IgAVN with nephrotic proteinuria was pathologically relatively severe. The majority of children with ISKDC grade VI IgAVN presented with nephrotic syndrome with hematuria [41]. However, patients with mild proteinuria sometimes had severe kidney histological impairment [37].

Xu et al. explored the utility of the Oxford classification in IgAVN in Chinese children. A total of 104 children with IgAVN were included and pathologically reclassified according to the Oxford classification scoring approach [10]. The percentages of M1, E1, S1, T1/2, and C1/2 were 66%, 56%, 62%, 58%, and 61%, respectively. Mesangial hypercellularity (M1) was shown to be highly associated with proteinuria (*P* = 0.019). At the time of biopsy, tubular atrophy/interstitial fibrosis (T1/2) and crescent development (C2) were associated with a reduced estimated glomerular filtration rate (eGFR) (*P *= 0.002 and *P *= 0.038, respectively). S1 was highly related to the main outcome of decreased kidney function in univariate time-dependent analysis (*P *= 0.025). However, T1/2 was strongly associated with proteinuria remission (*P *= 0.035). The Oxford classification system seems to be valid for Chinese children with IgAN, according to these results.

Generally, the severity of proteinuria and kidney function in Chinese children with IgAVN is comparable to that of children in the USA and Japan (Supplementary Tables 1 and 2) [34, 35]. Crescents were more prevalent in Chinese children (60–80%) than in Japanese children with IgAVN (1.9%) [34].

Studies have also compared the pathological patterns of IgAN and IgAVN. Over the course of more than a year, it was reported that M1 is more prevalent in IgAN, but IgAVN had more S1 than IgAN. These findings illustrate the pathophysiological differences between IgAN and IgAVN [30]. In Chinese children, considerable clinical and pathological distinctions between IgAVN and IgAN have been documented, contradicting the universal disease entity theory [42].

## Long-term prognosis of IgAN and IgAVN in Chinese children

The results from pediatric patients with IgAN tended to be better than those described in adults [29]. At a median follow-up of 86.8 months, a single site cohort study found that the five-, ten-, and fifteen-year kidney survival rates were 95.3%, 90.3%, and 84%, respectively [9]. Le et al. [7] reported that during a median 56-month follow-up period, 12.4% of children with IgAN had kidney failure or a 50% reduction in kidney function. Tubular atrophy/interstitial fibrosis was shown to be the only factor that was independently associated with poor kidney outcomes. In another trial, 14% of children reached the combined endpoint, which was defined as a 50% reduction in estimated glomerular filtration rate (eGFR) or kidney failure, after being followed for a median of 7.2 (4.6–11.7) years. Long-term kidney prognostic variables, such as S1 and T1/2 lesions, were shown to be beneficial [8]. Despite the pathological classification, some laboratory parameters were considered to be potential predictors for long-term prognosis. Proteinuria remission was crucial for improving kidney prognosis in children with IgAN and nephrotic-range proteinuria [29]. When compared to the full remission group, the probability of kidney function worsening was considerably greater in the partial remission and no response groups [29]. A retinol-binding protein (RBP) level in the urine of less than 0.7 g/ml might also indicate a poor kidney outcome [9]. The urinary RBP level is a marker of tubulointerstitial damage, and tubulointerstitial involvement is associated with the prognosis of IgAN.

The prognosis for Chinese children with IgAN is comparable to that of Japanese and European children. In the VALIGA European cohort, the 15-year survival rate free from the combined events of a 50% decline in eGFR or kidney failure was 93.7% in children with IgAN [5]. In Japan, 11.3% of children with IgAN progressed to abnormal kidney function after a median follow-up duration of 9.9 (7.4–13.3) years (Supplementary Tables 1 and 2) [33].

Barbour et al. developed and validated international risk prediction models in adults with IgAN using a wide multinational group of datasets with various ethnic representations [43]. Using existing clinical and laboratory risk indicators and the MEST score, the models were shown to be accurate for predicting disease progression and patient risk stratification in IgAN in multiethnic populations. After that, they used a multiethnic international cohort of 1060 children with IgAN, including 422 Chinese children, to update the prediction tool for use in children. Eventually, age, eGFR, proteinuria, mean arterial blood pressure (MAP), use of RASB at biopsy, race/ethnicity, use of immunosuppression prior to biopsy, and Oxford pathological scores were chosen as predictors in the models [31]. The models were considered to be accurate and validated methods for disease prediction and risk stratification in multiethnic IgAN.

It is generally considered that the prognosis of IgAVN is relatively good. In a study of 104 Chinese children diagnosed with IgAVN, 58.7% of all patients showed complete remission, 77.9% achieved proteinuria remission, and 36.5% experienced recurrence of nephritis [10]. During a 40-month (12–145) follow-up period, 7.7% of patients had impaired kidney function, as defined by a 50% drop in baseline eGFR or an eGFR of less than 90 ml/min per 1.73 m^2^. eGFR and proteinuria at the time of biopsy were shown to be substantially linked with decreased kidney function in multivariate analysis. Lin et al. retrospectively compared children and adults admitted to the hospital with the diagnosis of IgAV [15]. Adults had more frequent and severe kidney involvement and considerably greater hypertension and proteinuria. The overall prognosis was good in both age groups in China. Coppo et al. reported that 7.2% of Italian children with IgAVN progress to CKD stage 5 after 4.5 to 12.0 years [44]. This outcome was similar to that from the data of Chinese children. Further studies exploring the long-term prognosis and risk factors of this disease are still needed.

## Guidelines and real-world practice for pediatric IgAN and IgAVN in China

In China, during recent years, pediatric nephrologists have struggled to increase the quality of care and standardize the diagnosis and treatment of children with kidney diseases. Several decades ago, biopsy was not necessary for the diagnosis of IgAN. In 2000, a proposal for the clinical classification, diagnosis, and treatment of childhood glomerular diseases was formulated by the Chinese Society of Pediatric Nephrology (CSPN) [45]. It explicitly states that kidney pathology is pivotal for IgAN diagnosis. Since 2007, a series of guidelines on the diagnosis and treatment of childhood kidney diseases have been developed following the principles of evidence-based medicine by a group of well-trained pediatric nephrologists from the CSPN [16, 17, 46, 47]. The guidelines are supposed to provide more comprehensive and reliable references for pediatricians in clinical practice.

It is worth noting that a large difference exists between Chinese evidence-based guidelines [16, 46] and the KDIGO guidelines [11] regarding the treatment of pediatric IgAN. According to the Chinese guidelines [16], corticosteroids plus immunosuppressive agents are suggested to be used in cases with nephrotic proteinuria, moderate to severe mesangial proliferation, or crescent formation. However, only one randomized controlled trial (RCT) from Japan supported the combination treatment of prednisolone and azathioprine in childhood IgAN with diffuse mesangial proliferation [48]. In another RCT from Japan, early treatment with steroids is considered effective in adulthood IgAN with sub-nephrotic proteinuria and diffuse mesangial proliferation [49]. Steroids are recommended by the Chinese guidelines to start at a dose of 1.5–2 mg/kg/day for 4 weeks, followed by alternative daily dose tapering over 1–2 years. Methylprednisolone pulse is suggested in cases of crescents with more than 25% of glomeruli. In the Chinese guidelines [16], regarding immunosuppressive agents, cyclophosphamide is the first-line choice in children with daily proteinuria higher than 50 mg/kg or moderate to severe mesangial proliferation. Cyclophosphamide (intravenous or oral) is also indicated in children with crescent formation with more than 25% of glomeruli. However, there was only one prospective open-labeled trial supporting the use of steroids and intravenous cyclophosphamide in adults with crescentic IgAN [50]. Azathioprine or mizoribine combined with heparin, warfarin, and dipyridamole was also mentioned for pediatric IgAN with nephrotic proteinuria. There is scarce evidence for leflunomide or mycophenolate. By contrast, in the 2021 KDIGO guidelines [11], no specific recommendation is given for the use of steroids and immunosuppressive agents in children with IgAN. It mentions that the approach of glucocorticoids plus nonglucocorticoid immunosuppressants may be considered in more severe cases. For the choice of immunosuppressants, only cyclophosphamide is considered in children with rapidly progressive IgAN, with limited evidence.

Chinese evidence-based guidelines appear to be more aggressive with respect to the indication of steroids and immunosuppressive agents. This is consistent with real-world practice in China. In 2007 and 2013, two national surveys were conducted by the CSPN in China, and these surveys included 1417 and 1349 children diagnosed with IgAN, respectively [1, 27]. In practice, steroids were prescribed in 42.2 to 49.5% of children with IgAN. Cyclophosphamide was the most prevalently used nonglucocorticoid immunosuppressant, and it was prescribed in 12.1 to 19.6% of cases. The proportion of MMF increased to 6.3% in the 2013 survey. It is worth noting that approximately 20% of pediatric IgAN patients were treated with Chinese herbal medicine. However, there is little evidence to support the use of herbal medicine. The Chinese evidence-based guidelines on childhood IgAN in 2016 have no recommendation for herbal medicine. Tonsillectomy was uncommon, occurring in fewer than 1% of cases. Furthermore, for children with nephrotic proteinuria, steroids, CTX, and MMF were intended to be used in nearly 80%, 40%, and 10% of cases, respectively. It should be noted that half of the patients with massive proteinuria did not receive any ACEI or ARB medication, deviating from guideline recommendations. Steroids were administered in 13.8% and 17.6% of cases with isolated hematuria during the periods of 2008–2011 and 1995–2004, respectively. Chinese clinicians were more inclined to prescribe steroids or immunosuppressants in IgAN children compared to that as recommended by the guidelines. However, the outcome of IgAN in children is similar in China, Japan, and Europe. Further study is still needed to explore the necessity of “aggressive treatments.”

In China, an evidence-based guideline on the diagnosis and treatment of IgAVN was published in 2017 [17]. Steroids alone or in combination with immunosuppressive agents are suggested for children with proteinuria levels greater than 1 g/day/1.73 m^2^ or mesangial proliferation. For children with nephrotic proteinuria, hematuria and proteinuria, or crescent formation, steroids plus cyclophosphamide are recommended. Methylprednisolone pulse therapy may be used in cases with diffuse mesangial proliferation or crescents in more than 50% of the glomeruli. In addition, regimens of steroids plus cyclosporine, mycophenolate, azathioprine, mizoribine, or leflunomide are also described in Chinese guidelines. For rapidly progressive cases, cases with crescents in more than 75% of glomeruli, or membranoproliferative-like lesions, methylprednisolone pulse therapy is recommended, followed by a combination of oral steroids combined with cyclophosphamide, heparin, and dipyridamole. Compared to the KDIGO guidelines, more immunosuppressive agents are suggested by Chinese evidence-based guidelines.

In 2013, a national survey reviewed a total of 4863 children diagnosed with IgAVN in 40 centers across China [37]. Treatment with steroids or steroids combined with immunosuppressive agents was recorded in 75.6% of children. The most widely used regimen was steroids alone (34.0%). Regarding immunosuppressive agents, cyclophosphamide was the most prevalent (8.5%). This was followed by mycophenolate (8.3%), leflunomide (5.1%), tacrolimus (3.4%), and cyclosporine (3.1%). It is worth noting that nearly half of the patients with isolated hematuria received steroids or immunosuppressants. In addition, 35.9% of cases with hematuria and proteinuria are treated with steroids only. Patients with crescent formation were mainly treated with steroids combined with cyclophosphamide and methylprednisolone. After the first guidelines were published in 2009 in China, the rate of steroids plus cyclophosphamide increased from 6.6 to 16.5% in cases with nephrotic proteinuria. This implies the influence of guidelines on real-world practice. However, there was no unified treatment protocol with respect to steroids and immunosuppressants. High diversity has been observed in the treatment approaches of IgAVN in China. Further prospective analysis or multicenter randomized controlled trials are still needed to identify the optimal treatment.

In summary, the clinical manifestations and pathological features of IgAN and IgAVN are variable in Chinese children. Generally, the severity of proteinuria, kidney function and blood pressure in Chinese children with IgAN or IgAVN is comparable to that of children with IgAN or IgAVN in Europe, the United States and other Asian regions, such as Japan. Compared to Caucasian children and Japanese children, crescents were more common in Chinese children with IgAN. Chinese children with IgAN or IgAVN might be more inclined to be treated with steroids or immunosuppressive agents. Eventually, approximately 10% of Chinese children with IgAN or IgAVN progress to abnormal kidney function or kidney failure. Unfortunately, there is very little biomedical literature to support treatment strategies in children with IgAN. Since January 2016, a multicenter registry of IgAN in Chinese children, named the “Registry of IgA Nephropathy in Chinese Children” (Clinical Trials.gov NCT03015974), has been developed to collect data to guide therapy for these individuals. Children with IgAN were recruited from 30 different sites throughout China. Demographic, phenotypic, therapeutic, and outcome data and biospecimens were prospectively collected (http://www.igaregister.com/). To date, more than 900 children with primary IgAN have consented, been enrolled, and followed up. Further studies exploring the precise treatment regimens for childhood IgAN or IgAVN are still needed in the future.

## Supplementary information

Below is the link to the electronic supplementary material.

Supplementary file1 (DOCX 26 kb)

Supplementary file2 (DOCX 18 kb)
