# Supplementary material for: Diagnosis and treatment of IgA nephropathy and IgA vasculitis nephritis in Chinese children
Source: Pediatr Nephrol. 2022 Nov 8;38(6):1707–15. doi: 10.1007/s00467-022-05798-6 (PMC10154255; doi:10.1007/s00467-022-05798-6)
Supplement: Supplementary file 1 — Supplementary file1 (DOCX 26 kb) [file 467_2022_5798_MOESM1_ESM.docx]

| Supplementary Table 1 Clinical findings at biopsy, pathological classification, and follow-up in children with IgAN | | | | | | | | | | | | | | | | | | | |  |
| --- | --- | --- | --- | --- | --- | --- | --- | --- | --- | --- | --- | --- | --- | --- | --- | --- | --- | --- | --- | --- |
| Reference | N | Study type and setting | Inclusi on/exclusion criteria | Study period | Age, years old | Male, % | Race/ethnicity | Clinical findings | | | Pathology classification | | | | | Treatment | Follow up, months | Prognosis | Significant predictors for outcomes | |
|  |  |  |  |  |  |  |  | eGFR, ml/min/1.73m2 | Proteinuria | MAP, mmHg | M1, % | E1, % | S1, % | T1/T2, % | C1/C2, % |  |  |  | Predictors | Outcomes |
| The Subspecialty Group of Nephrology Society of Pediatrics, Chinese Medical Association[1] | 1349 | A national retrospective survey from 33 hospitals | Diagnosed as IgAN, age ≤14 years old, hospitalized children. IgAV, lupus and chronic liver disease were excluded. | 1995-2004 | 8.5±3.1 | 67.4 | Chinese | Isolated hematuria, 41.2% Isolated proteinuria, 1.9% Acute glomerulonephritis, 10.1% Nephrotic syndrome, 23.8% Rapid progressive glomerulonephritis, 1.3% Chronic glomerulonephritis, 1.0% | | | By Lee's grading Grade I, 14.8% Grade II, 28.5% Grade III, 41.4% Grade IV, 13.8% Grade V, 1.5%a | | | | | GC, 42.2% CTX, 12.1% RASB, ACEI, 31.6% Tonsillectomy, 7.5% | 24.4 (range 0.5-120.0) | Disease deterioration, 2.22% | NA | NA |
| Working Group for National Suvery on Status of Diagnosis and Treatment of Childhood Renal Diseases[27] | 1417 | A national retrospective survey from 35 hospitals | Diagnosed as IgAN, age ≤18years old. IgAV, lupus and chronic liver disease were excluded. | 2008-2011 | 10.0 (range 0.5-18.0) ^b^ | 67.2 | Chinese | Isolated hematuria, 15.8% Isolated proteinuria, 0.8% Hematuria and proteinuria, 37.0% Acute glomerulonephritis, 12.7% Nephrotic syndrome, 30.6% Rapid progressive glomerulonephritis, 1.3% Chronic glomerulonephritis, 1.8% | | | NA | NA | NA | NA | NA | GC, 47.5% CTX, 19.6% MMF, 6.3% IEF 1.7% RASB, 49.5% Tonsillectomy, 0.3% | NA | NA | NA | NA |
| Le et al.[7] | 218 | A multicenter validation study of the Oxford classification from 7 kidney centers | Biopsy-proven IgAN, age <18 years old, estimated GFR ≥30 ml/min/1.73m2, proteinuria ≥0.5g/day, total number of glomeruli ≥10. Secondary cause of mesangial IgA deposite were excluded. | NA | 14.0 (range 2.0-17.9) | 65.0 | Chinese | 134±42 | 1.5 (range 0.5-8.0) g/day | 88±11 | 45 | 23 | 62 | 7 | 44 | IS, 56.0% GC, 51.0% RASB, 61.5% | 56 (range 12-182) | 50% decline in eGFR or ESKD, 12.4% | T1/2 score | A 50% reduction in renal function or ESKD |
| Wu et al.[28] | 98 | A retrospective cohort study from single center | Biopsy-proven IgAN, age <18 years old, total number of glomeruli ≥8, serum C3 levels available, follow-up ≥12months. Secondary cause of mesangial IgA deposite were excluded | 2014-2018 | 9.7 (IQR 6.6, 12.2) | 67.3 | Chinese | 108.5±37.1 | 27.6 (IQR 11.5, 83.2) mg/kg/day | 83 (IQR 79, 89) | 42.9 | 70.4 | 18.4 | 21.4 | 65.3 | RASB, 78.6% GC, 79.6% Other IS, 60.2% | 25 (IQR 18, 36) | 30% reduction in eGFR, 5.1% ESKD, 1% Combine outcomes, 6.1% | Mesangial staining of C3 ≥ 2+ and blood C3 decreased | A 30% decline in eGFR or kidney failure |
| Zhao et al.[42] | 90 | A retrospective observational from single center | Biopsy-proven IgAN, nephrotic-range proteinuria, total number of glomeruli ≥8. Congenital kidney disease, secondary IgAN, hypertension and hyperuricemia were excluded. | 2011-2017 | 8.4±3.2^b^ | 71.1 | Chinese | 142.6±38.9 | 129.3±64.9mg/m2/h | NA | 97.8 | 6.7 | 47.8 | 4.4 | 25.5 | GC alone, 25.3% GC + IS, 72.2% | 45 (range 6-104) | Complete proteinuria remission, 88.6% Partial proteinuria remission, 10.1% No proteinuria remission, 1.3%c | Partial proteinuria remission group and no proteinuria remission | Renal function deterioration |
| Wu et al.[8] | 1243 | A retrospective cohort study from single center | Biopsy-proven IgAN, age ≤18 years old, total number of glomeruli ≥8, estimated GFR≥15 ml/min /1.73m2, follow-up ≥12months. Secondary cause of mesangial IgA deposite were excluded. | 2000-2017 | 14±4 | 68.0 | Chinese | 102±20 | 0.6 (IQR 0.3, 1.4) g/day/1.73m2 | 89±16 | 29.0 | 35.0 | 37.0 | 27.3 | 48.6 | RASB, 70% GC, 45% GC+Other IS, 19% | 86.4 (IQR 55.2, 140.4) | ESKD, 6.6% 50% reduction in eGFR, 7.2% Combined outcomes (either ≥50% reduction eGFR or ESKD or death), 14% | S score, T score C score in cases without immunosuppressive treatment | Either ≥50% reduction eGFR or ESKD or death. |
| Wu et al.[9] | 1243 | A retrospective study from single center | Childeren with biopsy-proven IgAN, total number of glomeruli ≥10, estimated GFR＞15 ml/min /1.73m2, follow-up ≥12months. Secondary or hereditary glomerulopathy were excluded. | 2000-2017 | 13.7±3.7 | 67.7 | Chinese | 102.1±19.8 | 1.0 (IQR 0.5, 2.4) g/day | 89.4±16.1 | 29.0 | 34.7 | 36.5 | 26.8 | 48.2 | RASB, 69.8% GC, 44.8% GC+Other IS, 18.6% | 86.8 (IQR 54.7, 140.2) | ESKD, 6.46% Combined event (either ≥50% decline in eGFR or ESKD or death), 7.2% The 5-, 10- and 15-year cumulative kidney survival rates, were 95.3%, 90.3% and 84%, respectively. | MBP, uric acid, eGFR, urine proteinuria, urine C3, urine RBC, S score, T score | Either ≥50% reduction eGFR or ESKD or death. |
| Barbour et al. [33] | 1060 | A multiethnic international cohort, including 14 cohorts | Biopsy-proven IgAN, age <18years old at kidney biopsy, available eGFR measurements. ESKD was excluded. | NA | 12.7 (IQR 9.6, 15.4) | 64.8 | Caucasian, 31% Japanese, 21% Chinese, 40.1% Other, 7.9% | 98 (IQR 79, 118) | 1.2 (IQR 0.5, 3.0) g/day/1.73m2 | 85.1 (IQR 77.3, 92.9) | 52.0 | 39.2 | 51.0 | 14.8 | 43.1 | RASB, 63.4% IS, 58.1% | 46.8 (IQR 25.2, 76.8) | eGFR loss, -2.34 ml/min/1.73m2/year 50% decline in eGFR or ESKD, a 5-year risk of 2.9% 30% decline in eGFR or ESKD, a 5-year risk of 9.1% | Age, eGFR, proteinuria, MAP, use of RASB at biopsy, race/ethnicity, use of IS prior to biopsy, and Oxford pathological scores | A 30% decline in eGFR or ESKD |
| Li et al. [40] | 142 | A retrospective study from single center | Diagnosed with IgAN by biopsy, age <18 years. | 2013-2018 | 10.1±3.0 | 59.2 | Chinese | 109.4 (IQR 88.8, 125.8) | 28.5 (IQR 11.9, 82.0) mg/kg/day | NA | 98.5 | 78.7 | 17 | 18.4 | 70.9 | NA | NA | NA | NA | NA |
| Coppo et al. [4, 5] | 174 | A multicenter retrospective study from 13 European countries, VALIGA European cohort (sub-Cohort 2) | Primary IgAN, aged <18 years, follow-up >1 year or progression to ESKD in <1 year. IgAVN, chronic hepatitis, diabetes or cancer were excluded. | NA | 12.7±3.6 | 71.8 | European | 117.0 (IQR 96.2, 120.0) | 0.8 (IQR 0.3, 2.2) g/day/1.73m2 | 87.5±11.4 | 21.8 | 13.8 | 42.5 | 6.3 | 14.9 | RASB, 66.7% CS/IS, 50.6% | 55.6 (IQR 29.8, 88.2) | Time-averaged proteinuria ≤0.5 in patients with baseline proteinuria >0.5 g/day/1.73 m2, 7.54% ESKD, 4.0% 50 % loss of initial eGFR, 4.6% Combined endpoint, 6.3% 15-year survival free from combined event (50% decline in eGFR or ESKD), 93.7% | Proteinuria and blood pressure during follow-up | eGFR slope |
| Matsushita et al. [29] | 53 | A retrospective cohort study from single center | Diagnosed as IgAN, age ≤15 years old. followed up for >5 years. | 1994-2007 | 12.0±3.6 | 58.5 | Japanese | 105.5±25.9 | >0.5g/gcr in 69.8% of cases | NA | Ratio of mesangial hypercellularity, 52.7±19.4% Endocapillary cellular proliferation, 18.9% Segmental sclerosis, 56.6% Tubular atrophy/ interstitial fibrosis, 0% Crescent formation, 28.3%a | | | | | NA | 118.8 (IQR 88.8, 159.6) | Non-remissiond, 49.1% eGFR, 109.7±26.7ml/min/1.73m2 eGFR <90 ml/min/ 1.73 m2, 11.3% ESKD, 0% | Proteinuria, particularly <0.5 g/gcr at 2 years | Remission at the last observationd |
| Shima et al. [31] | 161 | A retrospective study | Newly diagnosed IgA nephropathy. | 1977-1989 | 11.7 (range 3.6-19.4) | 63.0 | Japanese | 103±30 | 0.7 (range 0.0-13.7) g/day/1.73m2 | 79±11 | 0.49e | 0.8e | 13.1e | 3.3e | 9.2e | No treatment, 53% IS, 16% CS, 16% | 54 (range 12-170) | Proteinuria (>1g/d/1.73m2 or >1g/gcr), 8% eGFR <60 mL/min/1.73 m2, 4% ESKD, 0.6% | Mesangial hypercellularity, tubular atrophy/interstitial fibrosis, crescents | eGFR<60 mL/min/1.73m2 |
| Komatsu et al. [30] | 803 | A cross-sectional study, the Japan Renal Biopsy Registry (J-RBR) | Registered in J-RBR system, registered in IgAN as the pathogenesis, age <18 years old. | 2007-2012 | 15 (IQR 12, 17) | 56.5 | Japanese | NA | 0.6±1.1g/day 0.9±1.5g/gcr | NA | Mesangial proliferative GN, 93.2% Endocapillary proliferative GN, 0.7% Minor glomerular abnormality, 4.2% Focal segmental glomerulosclerosis, 0.1% Membranous nephropathy, 0.1% Membranoproliferative GN (type I and III), 0% Crescentic and necrotizing GN, 0.9% Sclerosing GN, 0.1%a | | | | | NA | NA | NA | NA | NA |
| Selewski et al. [32] | 173 | A longitudinal, prospective, observational study from 66 centers, Cure Glomerulonephropathy Network (CureGN) | Diagnosed with IgAN, with a diagnostic biopsy during the past 5 years, aged <18 years of age at biopsy. | NA | 12.5 (IQR 9.9, 15.2) | 61.8 | White, 82.4%, Hispanic/Latino, 12.2% | 98.6 (IQR 75.9, 122.0) | 1.1 (IQR 0.3, 2.3) g/gcr | NA | NA | NA | NA | NA | NA | CS, 46.2% CTX, 4.0% AZA, 5.2% MMF, 14.5% RASB, 67.1% | NA | NA | NA | NA |
| MAP, mean arterial pressure; eGFR, estimated glomerular filtration rate; NA, not available; GC, Glucocorticoid; CTX, cyclophosphamide; RASB, renin angiotensin aldosterone system blockade; MMF, mycophenolate mofetil; IEF, leflunomide; IS, immunosuppressive agent; IQR, interquartile range; CNI, calcineurin inhibitor; CI, confidence interval; AZA, azathioprine; GN, glomerulonephritis; ESKD, end-stage kidney disease; M1, mesangial proliferation; E1, endocapillary proliferation; S1, segmental sclerosis/adhesion lesion; T1/T2, moderate/severe tubular atrophy/interstitial fibrosis; C1/C2, crescent formation | | | | | | | | | | | | | | | | | | | | |
| aThe study did not present pathological data using the Oxford classification.  ^b^Age at disease onset. | | | | | | | | | | | | | | | | | | | | |
| cComplete remission was defined as the absence of proteinuria, disappearance of clinical manifestations, normalization of biochemical findings, and no worsening of kidney function. Partial remission was defined as a more than 50% reduction in proteinuria from baseline to 40mg/m2/h. No remission was defined as a less than 50% reduction or an increase in proteinuria or renal deterioration. | | | | | | | | | | | | | | | | | | | | |
| dRemission was defined as three consecutive negative results over a 6-month period in urinary occult blood tests, a urinary sediment red blood cell count of < 5 per high-power field, and UPCR of < 0.3 g/day (g/ g Cr).  eThe Oxford derivation cohort included children with proteinuria ≥ 0.5 g/day/1.73 m2. | | | | | | | | | | | | | | | | | | | | |
