# Supplementary material for: Diagnosis and treatment of IgA nephropathy and IgA vasculitis nephritis in Chinese children
Source: Pediatr Nephrol. 2022 Nov 8;38(6):1707–15. doi: 10.1007/s00467-022-05798-6 (PMC10154255; doi:10.1007/s00467-022-05798-6)
Supplement: Supplementary file 2 — Supplementary file2 (DOCX 18 kb) [file 467_2022_5798_MOESM2_ESM.docx]

| Supplementary Table 2 Clinical findings at biopsy, pathological classification, and follow-up in children with IgAVN | | | | | | | | | | | | | | | | | | | |  |
| --- | --- | --- | --- | --- | --- | --- | --- | --- | --- | --- | --- | --- | --- | --- | --- | --- | --- | --- | --- | --- |
| Reference | N | Study type and setting | Inclusion/exclusion criteria | Study period | Age, years old | Male, % | Race/ethnicity | Clinical findings | | | Pathology classification | | | | | Treatment | Follow up, months | Prognosis | Significant predictors for outcomes | |
|  |  |  |  |  |  |  |  | eGFR, ml/min/1.73m2 | Proteinuria | MAP, mmHg | M1, % | E1, % | S1, % | T1/T2, % | C1/C2, % |  |  |  | Predictors | Outcomes |
| Working Group for National Survey on Status of Diagnosis and Treatment of Childhood Renal Disease[35] | 4863 | A national retrospective survey from 40 hospitals | Diagnosed as IgAVN, hospitalized children. | 2008-2011 | 8.9±3.0^a^ | 60.4 | Chinese | Isolated hematuria, 13.2% Isolated proteinuria, 11.6% Hematuria and proteinuria, 58.2% Acute glomerulonephritis, 2.8% Nephrotic syndrome, 13.8% Rapid progressive glomerulonephritis, 0.2% Chronic glomerulonephritis, 0.2% | | | By ISKDC classification Grade I, 12.8% Grade II, 31.0% Grade III, 53.5% Grade IV, 2.3% Grade V, 0.3% Grade VI, 0.1%b | | | | | RASB, 48.9% GC, 58.0% CTX, 8.5% MMF, 8.3% CNI, 6.5% IEF, 5.1% | NA | NA | NA | NA |
| Xu et al. [10] | 104 | A retrospective study from single center | Diagnosed as IgAVN, age at renal biopsy < 18 years, total number of glomeruli ≥7, follow-up duration ≥ 12 months. Patient with comordid diseases were excluded. | 2003-2015 | 10 (range 4-17) | 56.0 | Chinese | 161±48 | 1.7 (range 0.1-10.8) g/day | 82±11 | 66 | 56 | 62 | 58 | 61 | GC, 93% Cytotoxic agents, 7% | 40 (range 12, 145) | 50% reduction in eGFR or eGFR < 90 ml/min/1.73 m2, 7.7% | eGFR and proteinuria at the time of biopsy, S1 by univariate time-dependent analyses | A 50% reduction in eGFR or eGFR <90ml/min/1.73m2 |
| Li et al. [40] | 57 | A retrospective study from single center | Diagnosed with IgAVN by biopsy, age <18 years. | 2013-2018 | 10.2±2.6 | 52.6 | Chinese | 114.2 (IQR 94.4, 131.1) | 24.2 (IQR 9.3, 60.8) mg/kg/day | NA | 92.7 | 81.8 | 23.6 | 10.9 | 80 | NA | NA | NA | NA | NA |
| Komatsu et al. [30] | 158 | A cross-sectional study, the Japan Renal Biopsy Registry (J-RBR) | Registered in J-RBR system, registered in IgAVN as the pathogenesis, age <18 years old. | 2007-2012 | 9 (IQR 6, 13) | 48.1 | Japanese | NA | 1.3±2.0g/day, 3.2±4.0 g/gcr | NA | Mesangial proliferative GN, 81.6% Endocapillary proliferative GN, 4.5% Minor glomerular abnormality, 9.5% Focal segmental glomerulosclerosis, 0% Membranous nephropathy, 0% Membranoproliferative GN (type I and III), 0.6% Crescentic and necrotizing GN, 1.9% Sclerosing GN, 0%b | | | | | NA | NA | NA | NA | NA |
| Selewski et al. [32] | 112 | A longitudinal, prospective, observational study from 66 centers, Cure Glomerulonephropathy Network (CureGN) | Diagnosed with IgAV, with a diagnostic biopsy during the past 5 years, aged <18 years of age at biopsy. | NA | 9.5 (IQR 6.9, 13.6) | 63.4 | White, 91.7% Hispanic/Latino, 10.7% | 109.5 (IQR 82.4, 127.7) | 2.1 (IQR 0.7, 5.0) g/gcr | NA | NA | NA | NA | NA | NA | CS, 73.2% CTX, 8.0% AZA, 8.9% MMF, 20.5% RASB, 62.5% | NA | NA | NA | NA |
| MAP, mean arterial pressure; eGFR, estimated glomerular filtration rate; NA, not available; GC, Glucocorticoid; CTX, cyclophosphamide; RASB, renin angiotensin aldosterone system blockade; MMF, mycophenolate mofetil; IEF, leflunomide; IS, immunosuppressive agent; IQR, interquartile range; CNI, calcineurin inhibitor; CI, confidence interval; AZA, azathioprine; GN, glomerulonephritis; ESKD, end-stage kidney disease; M1, mesangial proliferation; E1, endocapillary proliferation; S1, segmental sclerosis/adhesion lesion; T1/T2, moderate/severe tubular atrophy/interstitial fibrosis; C1/C2, crescent formation. | | | | | | | | | | | | | | | | | | | | |
| ^a^Age at disease onset.  bThe study did not present pathological data using Oxford classification. | | | | | | | | | | | | | | | | | | | | |
